# Eliapixant is a selective P2X3 receptor antagonist for the treatment of disorders associated with hypersensitive nerve fibers

**DOI:** 10.1038/s41598-021-99177-0

**Published:** 2021-10-06

**Authors:** Adam J. Davenport, Ioana Neagoe, Nico Bräuer, Markus Koch, Andrea Rotgeri, Jens Nagel, Alexis Laux-Biehlmann, Frederic Machet, Anne-Marie Coelho, Susan Boyce, Nikisha Carty, Mark J. Gemkow, Stephen D. Hess, Thomas M. Zollner, Oliver M. Fischer

**Affiliations:** 1 Discovery Chemistry, Dorothy Crowfoot Hodgkin Campus, Evotec UK, 114 Innovation Drive, Milton Park, Abingdon, Oxfordshire OX14 4RZ UK; 2 In Vitro Pharmacology, Manfred Eigen Campus, Evotec SE, Essener Bogen 7, 22419 Hamburg, Germany; 3 Pharmaceuticals Division, Drug Discovery, Candidate Generation & Exploration, Medicinal Chemistry, Bayer AG, Müllerstr. 178, 13353 Berlin, Germany; 4 Pharmaceuticals Division, Research and Development, Preclinical Research, Therapeutic Area Endocrinology, Metabolism and Reproductive Health, Bayer AG, Müllerstr. 178, 13353 Berlin, Germany; 5 Pharmaceuticals Division, Drug Discovery, Early Development, Drug Metabolism and Pharmacokinetics, Bayer AG, Müllerstr. 178, 13353 Berlin, Germany; 6 Research & Development, Pulmonary Drug Discovery Laboratory, Bayer US LLC, Boston, MA USA; 7 In Vivo Pharmacology, Campus Curie, Evotec SAS, 195 route d’Espagne, 31036 Toulouse, France; 8 In Vivo Pharmacology, Manfred Eigen Campus, Evotec SE, Essener Bogen 7, 22419 Hamburg, Germany; 9 Neurosciences, Manfred Eigen Campus, Evotec SE, Essener Bogen 7, 22419 Hamburg, Germany; 10 Present Address: Research, In Vitro Biology, Galapagos, Generaal De Wittelaan L11 A3,, 2800 Mechlin, Belgium; 11 Present Address: Life Sciences Chemistry, Nuvisan ICB GmbH, Müllerstr. 178, 13353 Berlin, Germany; 12 Present Address: Preclinical Compound Profiling, Nuvisan ICB GmbH, Müllerstr. 178, 13353 Berlin, Germany

**Keywords:** Reproductive disorders, Drug discovery, Pharmacology, Target validation, Ion channels in the nervous system

## Abstract

ATP-dependent P2X3 receptors play a crucial role in the sensitization of nerve fibers and pathological pain pathways. They are also involved in pathways triggering cough and may contribute to the pathophysiology of endometriosis and overactive bladder. However, despite the strong therapeutic rationale for targeting P2X3 receptors, preliminary antagonists have been hampered by off-target effects, including severe taste disturbances associated with blocking the P2X2/3 receptor heterotrimer. Here we present a P2X3 receptor antagonist, eliapixant (BAY 1817080), which is both highly potent and selective for P2X3 over other P2X subtypes in vitro, including P2X2/3. We show that eliapixant reduces inflammatory pain in relevant animal models. We also provide the first in vivo experimental evidence that P2X3 antagonism reduces neurogenic inflammation, a phenomenon hypothesised to contribute to several diseases, including endometriosis. To test whether eliapixant could help treat endometriosis, we confirmed P2X3 expression on nerve fibers innervating human endometriotic lesions. We then demonstrate that eliapixant reduces vaginal hyperalgesia in an animal model of endometriosis-associated dyspareunia, even beyond treatment cessation. Our findings indicate that P2X3 antagonism could alleviate pain, including non-menstrual pelvic pain, and modify the underlying disease pathophysiology in women with endometriosis. Eliapixant is currently under clinical development for the treatment of disorders associated with hypersensitive nerve fibers.

## Introduction

P2X3 receptors are adenosine triphosphate (ATP)-activated ion channels expressed on peripheral sensory neurons and are well-recognized players in the generation of pathological pain^1,2^. Studies have shown a reduced pain response in P2X3 receptor knockout mice^3–5^. Additionally, excessive activation of P2X3 receptors (e.g., due to the release of ATP from damaged or inflamed tissue), the upregulation of P2X3 receptors, or P2X3 receptor phosphorylation can contribute to neuronal hypersensitivity, which plays a role in chronic neuropathic and inflammatory pain syndromes^1,6^. As evidence, increased inflammatory pain sensitivity was found upon P2X3 receptor activation on afferent neurons in rat models of mechanical hyperalgesia^7^. Afferent neuronal hypersensitivity via P2X3 receptor signaling has also been implicated in pathways triggering bladder urgency^8,9^ and the cough reflex^10–14^. Therefore, inhibition of P2X3 receptors on peripheral sensory neurons could help in the treatment of several diseases associated with hypersensitive nerve fibers, including chronic pain, overactive bladder (OAB), and refractory and/or unexplained chronic cough (RUCC).

In addition to its role in neuronal hypersensitivity, it is hypothesized that excessive P2X3 receptor activation participates in a process termed neurogenic inflammation^6^. Neurogenic inflammation arises from the local release of inflammatory mediators by afferent neurons, including ATP, substance P, and calcitonin gene-related peptide (CGRP)^15–18^. In turn, these inflammatory mediators further activate local immune cells, creating a vicious cycle of activation and interplay of local immune cells and nerve fibers, which may also contribute to neuronal hypersensitivity^19,20^. However, whether P2X3 receptor antagonism can alleviate this cycle of neurogenic inflammation in vivo remains to be elucidated.

The discovery of nerve fibers in endometriotic lesions has led to intense research efforts on the potential underlying nerve fiber-associated mechanisms involved in endometriosis, including the role of neurogenic inflammation^19–23^. Endometriosis is a chronic, estrogen-dependent inflammatory disease characterized by the presence of ectopic endometrial tissue^24^. It affects ~ 5–10% of all women of reproductive age^25^ and considerably impacts quality of life^26,27^. The main symptoms of the disease are pain, including dysmenorrhea, non-menstrual pelvic pain, dysuria, dyschezia, and/or dyspareunia (i.e., painful intercourse)^28,29^. Many women also suffer from sub-fertility or infertility^28–30^. Suspected endometriosis is often treated initially with analgesics, combined oral contraceptives, or progestins^31–34^. When these treatments fail, surgical ablation of endometriotic lesions has become a commonly recommended treatment, yet a significant number of patients experience symptom recurrence^35^. Other second-line therapies aim to reduce systemic estrogen levels, but these can lead to menopausal symptoms, including loss of bone mineral density^33,36^. Therefore, new long-term treatment options for endometriosis that are effective and do not influence systemic hormone levels are required.

In women with endometriosis, ATP released during retrograde menstruation and due to mechanical stretch from endometriotic adhesions or fibrotic scar tissue could potentially activate P2X3 receptors, leading to neuronal hypersensitivity and pain^37–39^. Indeed, P2X3 receptor expression is elevated in ectopic endometrium in women with endometriosis^38,40^, with increased P2X3 expression observed in those experiencing pain^41^. Similarly, in rat models of endometriosis, the levels of endogenous ATP and expression of P2X3 receptors were increased in both endometriotic lesions and dorsal root ganglion (DRG) tissue, and delivery of a P2X3 receptor antagonist reduced peripheral hyperalgesia in this setting^17,37,42^. However, it remains unclear if the beneficial effects of P2X3 receptor antagonism on peripheral pain also translate to visceral pain (i.e., the disease-relevant pain pathway in women with endometriosis). Nonetheless, we hypothesize that directly targeting nerve fibers with P2X3 antagonists could reduce neuronal hypersensitivity, offering a targeted approach to treat endometriosis-associated pain. This strategy offers advantages over current hormone-based treatments for endometriosis (e.g., gonadotropin-releasing hormone agonists/antagonists^43^), as it would allow for long-term treatment, does not interfere with the menstrual cycle, and, importantly, could also target non-menstrual pelvic pain.

Based on its numerous clinical indications, including endometriosis, there is mounting therapeutic interest in identifying P2X3 receptor antagonists^44–46^. However, a major problem in developing P2X3 receptor antagonists is off-target effects, particularly changes in taste perception linked with blocking the P2X2/3 receptor heterotrimer^10,47,48^. P2X3 subunit-containing receptors are predominantly expressed on peripheral sensory neurons^1^, and P2X2 receptors are mainly expressed on sympathetic neurons^49^, while both P2X3 and P2X2 receptors are expressed on nerve fibers innervating the tongue^50^. As blocking the P2X3 receptor homotrimer alone can achieve anti-nociceptive efficacy^1^ and non-selective blockade of both P2X3 and P2X2/3 receptors leads to changes in taste perception in humans^10,47,48,51^, developing highly selective P2X3 receptor antagonists is key for their potential long-term safe and efficacious clinical use.

We have developed a selective P2X3 receptor antagonist, eliapixant (BAY 1817080; structure shown in Supplementary Fig. S1), which is currently undergoing clinical testing in phase IIb trials for RUCC (following promising results in a phase IIa study^52^), phase IIa trials for OAB, phase IIa trials for diabetic neuropathic pain, and phase IIb trials for endometriosis (Supplementary Table S1). Herein, we present the pharmacological characterization of eliapixant in vitro, including its potency, selectivity, and efficacy. We then examine the in vivo efficacy of eliapixant in animal models of inflammatory pain. Additionally, we provide the first in vivo experimental evidence that P2X3 receptor antagonism can inhibit the cycle of neurogenic inflammation. Finally, we test the effect of eliapixant on visceral pain (a more disease-relevant pain pathway) in an animal model of endometriosis-associated dyspareunia.

## Results

### Eliapixant is a potent P2X3 antagonist in vitro

**Figure 1:**
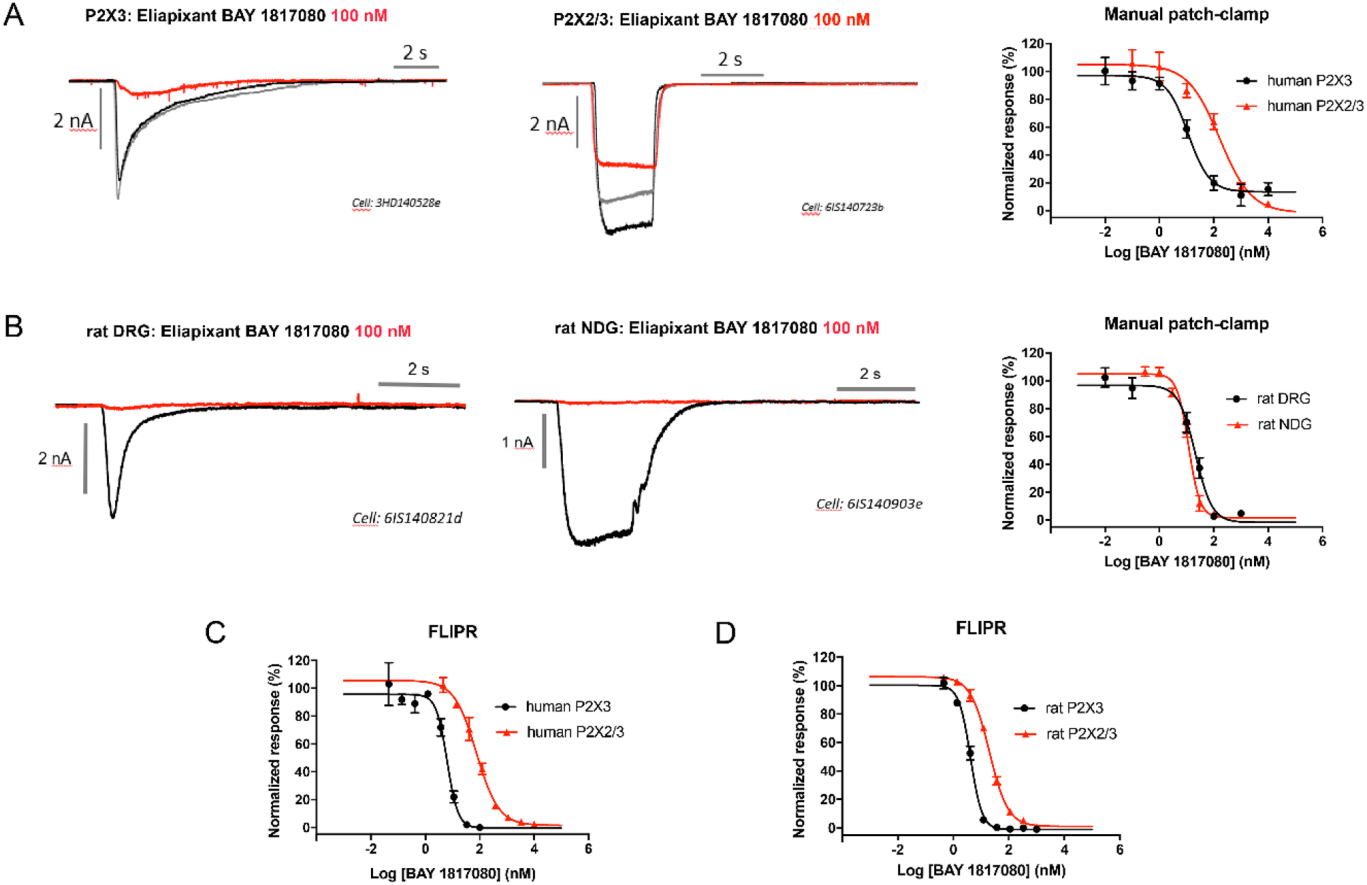
Eliapixant (BAY 1817080) is a potent antagonist of P2X3 in vitro. (**A**) Representative manual patch clamp traces for human P2X3 (left panel), human P2X2/3 (middle panel), and representative whole-cell patch-clamp concentration–response curves for eliapixant using recombinant cell lines expressing human P2X3 or P2X2/3. Responses to an EC_80_ concentration of α,β-meATP agonist (10 µM for hP2X3 receptors and 30 μM for human P2X2/3 receptors) are shown (human P2X3, n = 3–6 cells; P2X2/3, n = 3–10 cells). Data are expressed as the mean ± SEM. (**B**) Representative manual patch clamp traces for eliapixant against native rat dorsal root ganglion (DRG, L6–L4) neurons showing predominantly P2X3-like currents (left panel), and rat nodose ganglion (NDG) currents reflecting a majority of P2X2/3 channels (middle panel), dissected from female Sprague Dawley rats. Representative whole-cell patch-clamp concentration–response curves are shown in the right panel. Responses to the α,β-meATP agonist (10 µM) were measured (DRG, n = 3–6 cells; NDG, n = 3–5 cells). Data are expressed as the mean ± SEM. (**C**) Representative concentration–response curves for eliapixant against recombinant 1321N1 cell lines expressing human P2X3 homotrimers and human P2X2/3 heterotrimers in a fluorescence imaging plate reader (FLIPR) calcium-flux assay. Responses normalized to an EC_80_ concentration of the agonist, α,β-meATP, based on the increase in peak relative fluorescence units (RFU) are shown (mean ± standard error of the mean [SEM], n = 3). Representative FLIPR traces for human P2X3 and human P2X2/3 3 are shown in supplementary Fig. 2. (**D**) Representative concentration–response curves for eliapixant against recombinant HEK T-REx cell lines expressing rat P2X3 and rat P2X2/3 in a FLIPR calcium-flux assay (mean ± SEM, n = 3). Representative FLIPR traces for rat P2X3 and rat P2X2/3 are shown in supplementary Fig. 2.

Using recombinant 1321N1 cell lines and a fluorescence imaging plate reader (FLIPR)-based calcium flux assay, we showed that eliapixant was ~ 20-fold more potent against the human (h) P2X3 homotrimer receptor than the hP2X2/3 heterotrimer receptor. Specifically, the mean IC_50_ of eliapixant against the agonist α,β-meATP (a stable analogue of ATP) for hP2X3 receptors was 8 nM, whereas the mean IC_50_ against the agonist-mediated hP2X2/3 receptor response was 163 nM (Fig. 1A). Furthermore, eliapixant displayed high selectivity over other human P2X receptor subtypes: the mean IC_50s_ of eliapixant against recombinant cell lines expressing hP2X1 receptors, and the homotrimeric hP2X2, hP2X4, and hP2X7 receptors were 50 µM, 33 µM, 50 µM, and 50 µM, respectively. We confirmed this result by assessing eliapixant in a whole cell patch-clamp electrophysiological assay, also using α,β-meATP as the agonist. Here, eliapixant showed a ~ 13-fold greater potency for hP2X3 over hP2X2/3 receptors. The mean IC_50_ for the hP2X3 receptor was 10 nM versus 129 nM for the hP2X2/3 receptor (Fig. 1B). Considering the long half-life (> 24 h; data not shown) and corresponding low peak-trough ratio of eliapixant in humans, this selectivity for hP2X3 over the hP2X2/3 receptors should help alleviate unwanted off-target effects, like taste alterations.

Next, we investigated the activity of eliapixant against rat (r) P2X3 receptors, which shares 97% similarity and 93% identity homology with the human hP2X3 receptor. Eliapixant showed ~ sixfold more inhibitory potency against rP2X3 receptors (IC_50_ = 4 nM) than rP2X2/3 receptors (IC_50_ = 23 nM) in the FLIPR assay (Fig. 1C). We also assessed the inhibitory activity of eliapixant on native channels in a patch-clamp assay using neuronal tissues obtained from female Sprague Dawley rats. Eliapixant displayed potent antagonist activity against native channels from both DRG (predominately P2X3 receptors) and nodose ganglia (NDG, predominately P2X2/3 receptors) rat tissues, with IC_50_ values of ~ 19 nM and 12 nM, respectively (Fig. 1D). Our findings suggest that eliapixant is a potent inhibitor of rat P2X3 receptors in vitro.

### Eliapixant reverses hyperalgesia in rats and mice

Next, we assessed the in vivo efficacy of eliapixant in rats and mice using the Complete Freund’s Adjuvant (CFA)-induced mechanical hyperalgesia model of inflammatory pain. In the rat study, compounds were dosed orally 24 h after CFA intraplantar injection, and mechanical hyperalgesia was measured at 2, 4, and 6 h after administering the test compounds.

Eliapixant significantly reduced mechanical hyperalgesia in a dose-dependent manner, i.e., increased the Paw Withdrawal Threshold (PWTs) in response to mechanical pressure applied using a Pressure Application Measurement (PAM) apparatus. The effects of the low dose (1 mg/kg, n = 8) of eliapixant were moderate and statistically significant at 2 h post-treatment (*P* < 0.05). However, the effects of the medium and high doses (3 mg/kg and 10 mg/kg, n = 8 per dose group) were robust and significant at each time point of the study (*P* < 0.05 to 0.0001, Fig. 2A). We found that efficacy—defined as the reversal of hyperalgesia > 50%—was observed in the CFA model when measured plasma exposures corresponding to 80% receptor occupancy were achieved (data not shown). Thus, other in vivo models described in this study were dosed accordingly to ensure plasma exposures above this threshold.

**﻿Figure 2:**
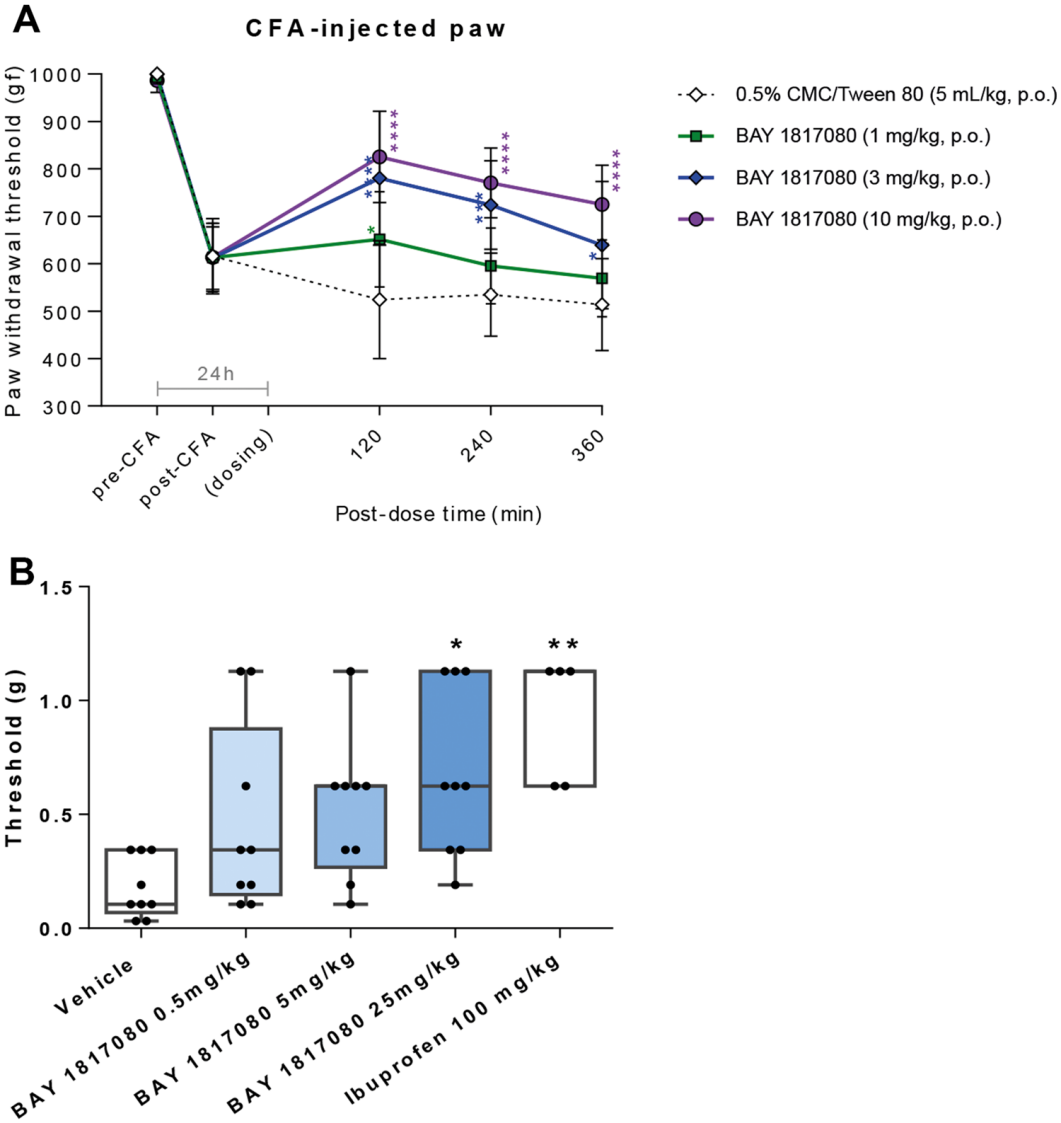
Eliapixant (BAY 1817080) reverses hyperalgesia in rodent models of inflammatory pain. (**A**) Dose-dependent efficacy of eliapixant on Complete Freund’s Adjuvant (CFA)-induced mechanical hyperalgesia in male Sprague Dawley rats (n = 8 per experimental group). All rats were orally administered with the test substances 24 h after CFA injection into one hind paw. Increasing pressure was applied to the hind paw until a behavioral response (paw withdrawal) was observed. The pressure at which the behavioral response occurred was recorded as the “Paw Withdrawal Threshold” (PWT), and was measured at 2, 4, and 6 h after test substance dosing. The mean ± standard deviation (SD) of the behavioral responses for the groups are shown; *P < 0.05, ***P < 0.001, ****P < 0.0001 versus vehicle (0.5% carboxymethylcellulose [CMC]:Tween 80), Dunnett’s post hoc test. (**B**) Dose-dependent efficacy of eliapixant on CFA-induced hyperalgesia in female C57BL/6 mice (n = 9 per experimental group). Compounds were administered orally, twice daily, starting one hour before CFA injection. Mechanical hyperalgesia was assessed using von Frey filaments, which were used to stimulate the hind paw. The strength of the von Frey filament used to stimulate the paw was expressed in [g], and the threshold was recorded when a response of the animal was observed. Response thresholds were determined 48 h after CFA injection. The mean ± SD of the behavioral responses for the groups are shown; *P < 0.05, **P < 0.001 versus vehicle (0.5% CMC:Tween 80), Dunnett’s post hoc test.

We confirmed our findings in a second species using a mouse model of hyperalgesia. In this model, mechanical hyperalgesia was induced in female C57BL/6 mice (n = 9) by injecting CFA into the plantar surface of one hind paw and assessed using von Frey filaments. The filaments were used to stimulate the hind paw, and a behavioral response was measured depending on the strength of the von Frey filament used (expressed in [g]). In addition, the treatment period was 48 h (repeat administration) in the mouse study compared with the relative short-term dosing (single administration) performed in the rat model. Compounds were administered orally, twice daily, starting one hour before CFA injection. Our results showed that eliapixant reversed hyperalgesia in the CFA-injected paw in a dose-dependent manner, with a minimal effective oral dose of 5 mg/kg, twice daily, at 48 h after CFA administration (Fig. 2B). Therefore, eliapixant shows significant efficacy in vivo in models of inflammatory pain, both in rat and mouse.

### Eliapixant reduces neurogenic inflammation in rats

**Figure 3:**
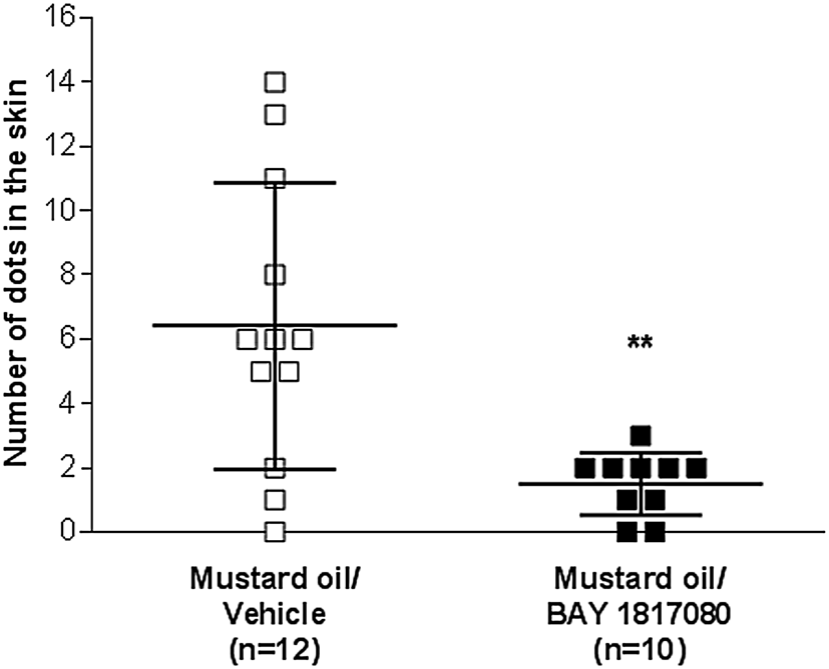
Eliapixant (BAY 1817080) shows efficacy in a rat model of uterine neurogenic inflammation. Female Sprague Dawley rats in pro-estrus phase were treated with either a bolus intravenous injection of eliapixant (0.7 mg/kg), which was followed by an intravenous infusion of 0.2 mg/kg eliapixant or the vehicle (saline) (n = 10 or 12, respectively). Inflammation of the uterus (induced by mustard oil) in rats results in blue dye extravasation (or blue dots) on the ventral and dorsal side of the abdominal region. The individual number of dots in the skin and the mean ± standard deviation for each group is shown; ** *P* < 0.01 versus the mustard oil/vehicle group, Mann–Whitney U test.

Next, we investigated the efficacy of eliapixant in a rat model of neurogenic inflammation, which is hypothesized to be an underlying pathology of endometriosis^19^. In this model, neurogenic inflammation was induced in anesthetized female Sprague Dawley rats by injecting mustard oil, an algogenic and sensitizing irritant, into the left uterine horn. Uterine inflammation leads to neurogenic plasma extravasation in the skin of the lower abdomen, which can be visualized via the injection of Evans blue dye^17^; the degree of inflammation can then be quantified by counting the number of blue dots. In our experiment, female rats were intravenously treated with eliapixant or a vehicle. Compared with the vehicle-treated rats (n = 12), rats treated with eliapixant (n = 10) showed a significantly reduced number of blue dots on the skin (*P* < 0.01; Fig. 3). To our knowledge, this is the first experimental evidence that P2X3 receptor inhibition can reduce neurogenic inflammation in vivo.

### P2X3 receptors are expressed in human endometriotic lesions

**Figure 4:**
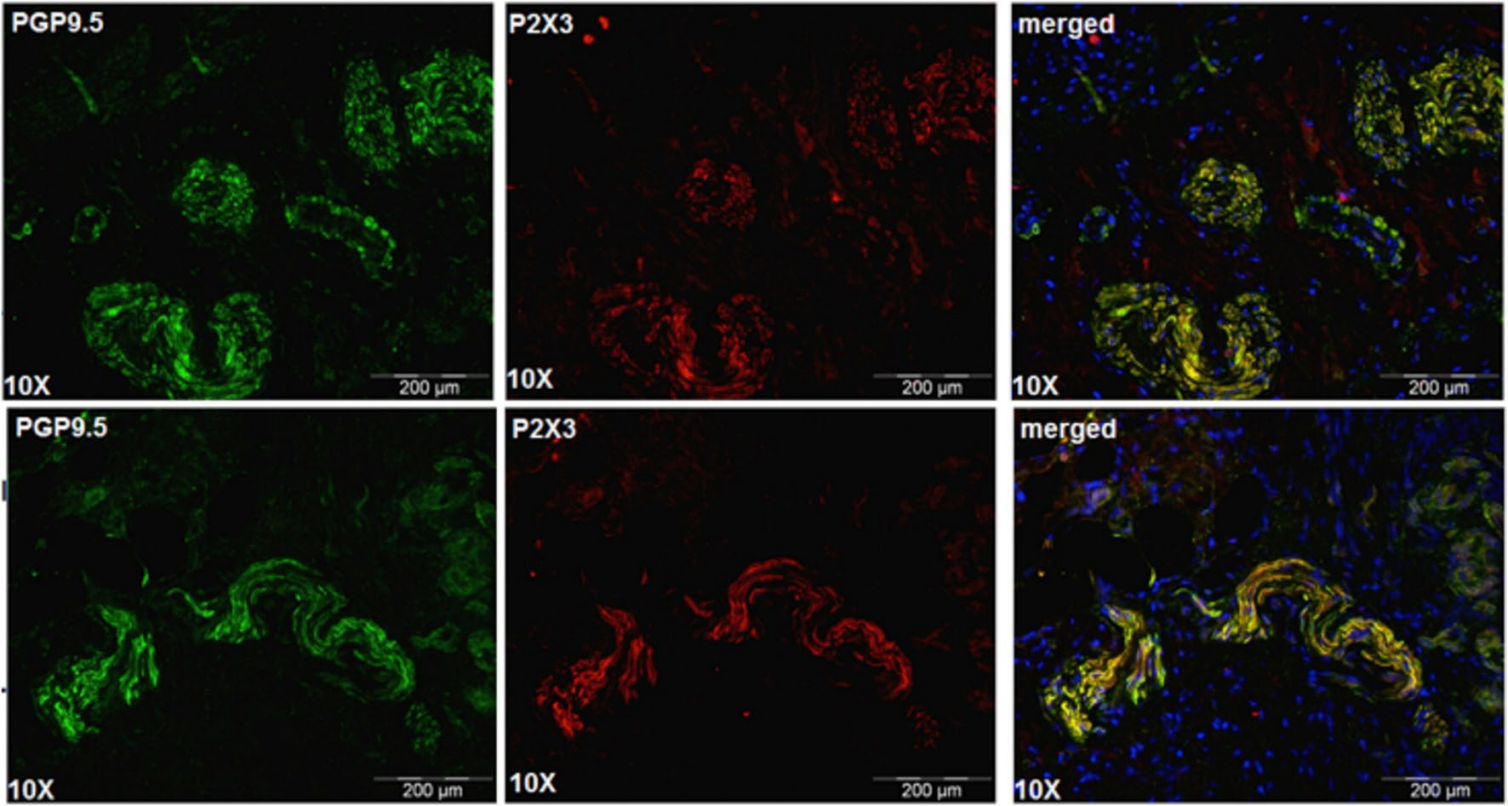
P2X3 is expressed on nerve fibers in human endometriotic lesions. Endometrial tissue samples were stained with antibodies for P2X3 and for the neuroendocrine marker, PGP9.5. Nerve fibers are stained green using PGP9.5 as a marker protein. P2X3 is stained red. The merged image shows the overlay of the two fluorescence signals showing P2X3 receptor expression (red) and the expression of PGP9.5 (green) which overlap spatially in the nerve fibers (yellow). Immunofluorescence images were obtained under a tenfold magnification.

Based on our findings regarding P2X3 and neurogenic inflammation, we hypothesized that eliapixant may be useful in the treatment of endometriosis. However, at the time of performing this study, there was no literature evidence of the presence of nerve fibers innervating endometriotic lesions, and the expression of P2X3 receptors on these nerves had not yet been shown. Therefore, first we confirmed the localization of these nerve fibers in different tissue samples of human endometriotic lesions using double immunofluorescence staining with an antibody for the detection of the neuroendocrine marker PGP9.5, and an anti-P2X3 receptor antibody used to detect P2X3 receptor expression (Fig. 4). These results established the presence of P2X3 receptors on nerve fibers innervating endometriotic lesions, and encouraged us to test eliapixant in preclinical models of endometriosis.

### Eliapixant reduces dyspareunia in rats

We examined the efficacy of eliapixant in a rat model of dyspareunia, which is often associated with endometriosis. This model was adapted from a rat model of endometriosis initially described by Berkley et al. in 2001^42^. In this model, dyspareunia was surgically induced in female Sprague Dawley rats by auto-transplanting small pieces of uterine horn onto mesenteric arteries of the small intestine and onto the wall of the distal colon. After 5 to 6 weeks, these uterine biopsies developed into vascularized cysts representing endometriotic lesion-like structures. The visceromotor response (VMR) to vaginal distension using an inflated vaginal balloon was determined by counting the number of abdominal muscle contractions via electromyography (EMG) in conscious animals as an objective measure of vaginal sensitivity. Eliapixant (or the vehicle) were dosed orally, twice daily, during two consecutive weeks, from week four to week five post-implantation of uterine horn tissue. The VMR response to vaginal distension was measured at week five (on-drug) and week six (one week off-drug) post-implantation of uterine horn pieces.

**Figure 5:**
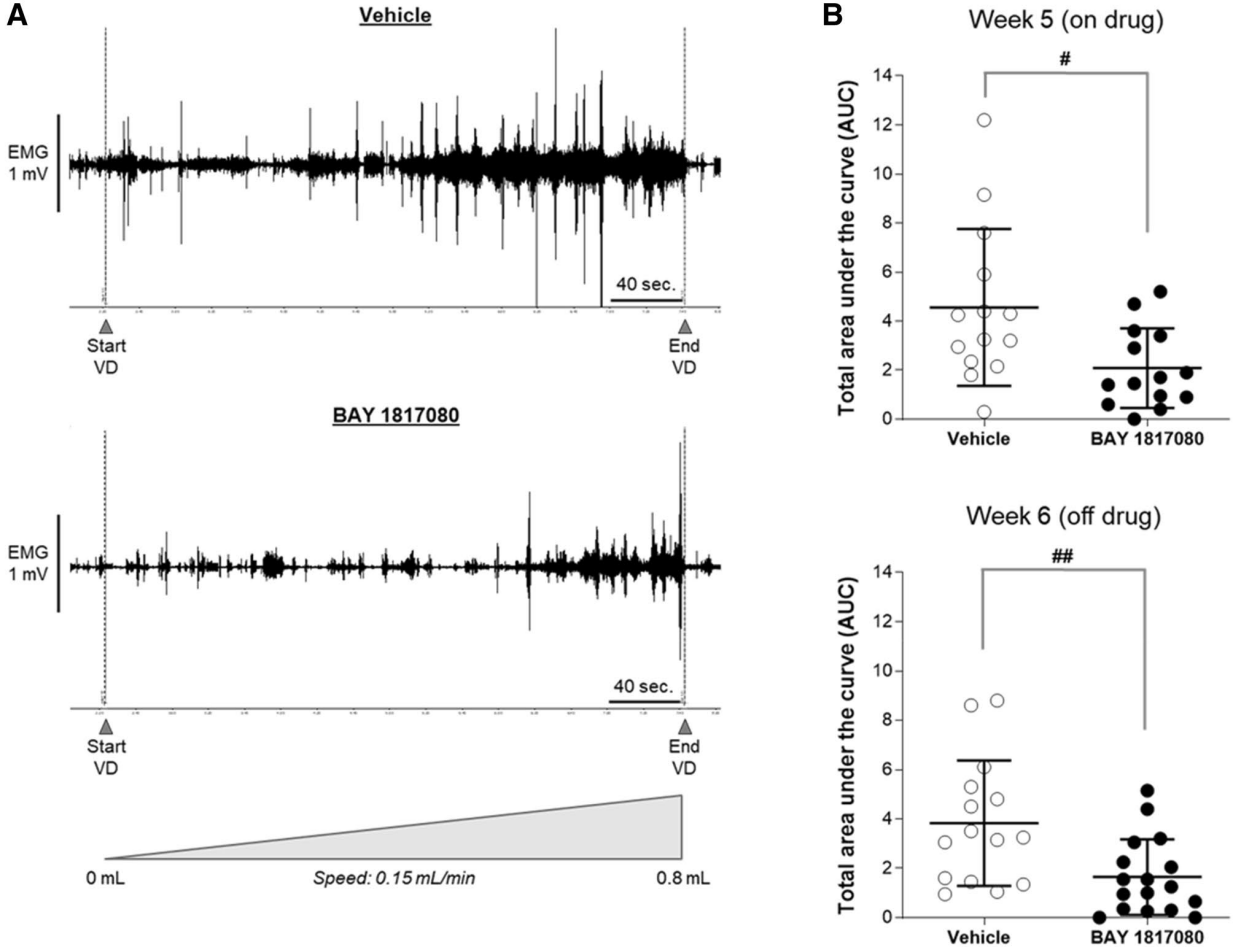
Eliapixant (BAY 1817080) shows efficacy in a rat model of dyspareunia. (**A**) In female Sprague Dawley rats, the visceromotor response (VMR) to vaginal distension (inflated vaginal balloon) was measured by counting the number of abdominal muscle contractions in conscious animals as an objective measure of vaginal sensitivity (individual records of VMR response to vaginal distension of one rat after vehicle and eliapixant, week six post-implantation). (**B**) Eliapixant (15 mg/kg) or the vehicle (0.5% carboxymethylcellulose [CMC]:Tween 80; 5 mL/kg) were dosed orally, twice daily in female rats (n = 15/17 per experimental group), during two consecutive weeks, from week four to week five post-implantation of the uterine horn pieces. VMR/vaginal distension tests were performed at week five (on-drug) and week six (off-drug) post-implantation when animals were in the pro-estrus phase. The individual area under the curve (AUC, determined from a plot of the cumulative number of abdominal contractions against vaginal distension volume for each animal) and the mean ± SD for each group is shown. One outlier was removed from the eliapixant treatment group at week five (Grubb’s test); ^#^P < 0.05, ^##^P < 0.01 versus vehicle (0.5% CMC:Tween 80), Mann–Whitney U test.

In vehicle-treated rats (n = 14), the cumulative number of abdominal contractions increased as a function of vaginal distension volume at week five and week six. This increase was associated with a significant rise in the corresponding area under the curve (AUC), which confirms the presence of vaginal hyperalgesia in these animals. Conversely, the eliapixant-treated rats (n = 15) presented a significantly decreased vaginal hyperalgesia (i.e., a decrease in the cumulative number of abdominal contractions or AUC) compared with vehicle-treated animals (Fig. 5). Furthermore, this decrease in vaginal hyperalgesia was not only observed while the animals were on drug treatment (5 weeks post-implantation; *P* < 0.05), but also when the animals were off drug treatment for 1 week (6 weeks post-implantation; *P* < 0.01; Fig. 5). Based on the half-life of eliapixant in rats (~ 2.8 h; data not shown), the drug concentration would be too low by this time point (i.e., 1 week off-drug) to remain efficacious.

## Discussion

P2X3 receptor antagonists have shown preclinical efficacy (or are undergoing clinical investigation) for treating conditions that have neuronal hypersensitivity as the commonality underpinning their pathophysiology, such as RUCC and pain disorders^53^. However, the broader and long-term usability of preliminary P2X3 receptor antagonists has been hampered by the lack of selective antagonists, which has led to tolerability issues with taste alterations^10,47,48^. Therefore, we developed a novel P2X3 receptor antagonist, eliapixant, to overcome the limited usability of existing molecules.

In this study, we showed that eliapixant has high in vitro potency (nM) and selectivity for human P2X3 receptors over P2X2/3 (~ 13–20-fold) and other human P2X receptor subtypes (> 6.50-fold over P2X1, P2X4, P2X7; > 4.125-fold over P2X2). We also proved that oral administration of eliapixant rapidly reduced mechanical hyperalgesia in CFA-induced inflammatory pain models in rodents. This effect was similar in both rat and mouse in vivo models, suggesting the mechanism is not species-dependent and supporting previous findings in P2X3 knockout mice as evidence for the role of P2X3 in inflammatory pain^3,4^.

Excessive P2X3 receptor activation is also hypothesized to contribute to neurogenic inflammation^6^, whereby the initial inflammatory response triggers a pain response and stimulates nerve fibers to release inflammatory mediators, which further activate the immune system and sensitize peripheral nerve endings^19,20,38^. We showed that eliapixant treatment resulted in a robust reduction of neurogenic plasma extravasation following uterine inflammation in rats, thus providing the first experimental evidence that P2X3 receptors contribute to or drive this pathogenic process. It is worth noting that substance P is the main neuropeptide contributing to this process in rats, while CGRP is the main contributor in humans; therefore, whether the effects observed with eliapixant in rats translate in humans requires further clinical assessment. Nonetheless, our findings substantiate the role of P2X3 receptors beyond that of nerve fiber signaling to immunomodulation for the first time.

Our findings led us to hypothesize that P2X3 receptor antagonists could specifically target this localized and self-perpetuating process of neurogenic inflammation in endometriosis (which can be triggered, for example, by retrograde menstruation). However, at the time of this study, there was no experimental evidence of the presence of nerve fibers in endometriotic lesions. Therefore, we investigated whether P2X3 receptors are expressed on nerve fibers innervating endometriotic lesions in patient tissue samples. Since conducting our experiments, the presence of nerve fibers in endometriotic lesions (and more specifically, the presence of peptidergic nerve fibers) has been confirmed^54^. Additionally, Ding et al.^38^ recently reported the expression of P2X3 receptors in endometriotic lesions, although they found that P2X3 receptors were highly expressed in endometrial epithelial cells rather than in nerve cells. In contrast, we found P2X3 receptors are mainly expressed in the peripheral nervous system, in line with previous reports on P2X3 receptor expression (reviewed by Ford^55^), and did not observe their expression in glandular epithelial cells. This difference in the distribution of P2X3 may reflect the different detection systems used in the study by Ding et al.^38^ compared to ours (i.e., different antibodies).

We then examined the long-term effect of eliapixant therapy in a rat model of dyspareunia (i.e., painful intercourse), a key symptom in endometriosis^28,29^. In this model, which was first described by Berkley et al.^42^, endometrial tissue is transplanted in an autologous manner onto mesenteric arteries of the small intestine and onto the peritoneum of rats, leading to increased vaginal hyperalgesia. This model provides a clear benefit over other models that focus only on peripheral hyperalgesia (as used recently by Yuan et al.^56^) and do not reflect visceral pain pathways, as the disease-relevant pain pathway in endometriosis. We found eliapixant significantly reduced vaginal hyperalgesia (dyspareunia) in this surgically-induced rat model of endometriosis, and the positive effect was sustained for a week beyond treatment cessation.

The sustained efficacy in the absence of eliapixant suggests that P2X3 receptor antagonism could be modifying the underlying disease, which is an exciting proposition for endometriosis treatment. The underlying mechanism by which eliapixant modifies disease pathology may involve interference with neurogenic inflammation, a hypothesis that awaits further experimental elucidation in preclinical and clinical studies. Moreover, eliapixant does not block the menstrual cycle, and could provide relief for women with non-menstrual pelvic pain. Thus, eliapixant treatment could simultaneously provide disease modification (e.g., slow disease progression), while also providing rapid and sustained pain relief (i.e., by blocking ATP-mediated nociception and reducing inflammatory pain) in women with endometriosis.

The high selectivity of eliapixant in humans combined with its long human half-life (and low peak-trough ratio) should alleviate off-target effects, thus enabling its long-term use (in contrast to the current treatment options for endometriosis). This has recently been verified in both a phase IIa and a Ph IIb study in patients with RUCC, where eliapixant was well tolerated with a low rate of mild taste-related adverse events (AEs)^52,57^. This is unlike that of the non-selective P2X3 and P2X2/3 receptor antagonist gefapixant, with an IC50 ratio of IC50(P2X3) vs. IC50(P2X2/3) of 1.5^51^. Gefapixant has been associated with a high incidence of taste-related AEs in phase II/III clinical trials for RUCC, with the latest phase III data reporting taste-related AEs in 58–69% of participants^10,58–61^.

In addition to gefapixant, other P2X3 receptor antagonists are currently undergoing development as potential treatments for conditions involving the P2X3 receptor. For example, S-600918 has been clinically shown to reduce cough with a low incidence of taste disturbance in patients with RUCC (n = 31)^62^. Similarly, BLU-5937 has demonstrated good potency and selectivity (P2X3 homotrimeric receptors IC_50_ = 25 nM and P2X2/3 heterotrimeric receptors IC_50_ > 24 μM) in addition to antitussive efficacy with a lack of taste disturbances in animal models^63^; however, it did not reach its desired primary endpoint in a recent phase IIa study in subjects with RUCC^64^ and clinical investigation is ongoing.

There are some limitations to our study. First, when considering our in vitro and in vivo data in rats, and in contrast to the in vitro effects in humans, eliapixant showed similar potency in native P2X3 and P2X2/3 receptors derived from primary rat DRG neurons and rat NDG neurons. Although this finding likely reflects the different distribution of P2X2 mRNA in rats compared to humans (i.e., there is a significant contribution of P2X2/3 in rat DRGs^65^ while the P2X3 homotrimer is the predominant channel in human DRGs), it does raise the question about which receptor type is the relevant one to target. However, a selective antagonist (AstraZeneca, AZ004) for rat P2X3 receptors (IC_50_ = 280 nM) over rat P2X2/3 receptors (IC_50_ > 2500 nM) has shown good efficacy in a rat model of CFA-induced rat model of hyperalgesia^66^, which supports our claim that the P2X3 homotrimer receptor is responsible for mediating inflammatory pain. In addition, we have shown that eliapixant has similar efficacy to the less selective P2X receptor antagonist, gefapixant, in a blocking activation of isolated human vagus nerves (published in Dicpinigaitis et al.^67^), supporting a major role of P2X3 receptors in this assay. As further evidence, both eliapixant and S-600918 have shown efficacy in treating RUCC patients in a phase IIa setting^52,62^, further confirmed in a Ph IIb trial with eliapixant^57^. Hence, it is likely that the P2X3 homotrimer receptor is also driving the observed beneficial effects of eliapixant in our rat models of inflammatory pain, neurogenic inflammation, and dyspareunia. Second, our immunofluorescence staining experiments to assess P2X3 receptor expression in human endometriotic lesions were based on only three human tissue samples, and thus, it is necessary to confirm the expression pattern of the P2X3 receptor using more samples. Finally, we used rodent models to evaluate the effect of P2X3 receptor antagonism in endometriosis, which is a non-menstruating species. Therefore, the efficacy of eliapixant in the treatment of endometriosis requires confirmation in the form of human clinical trials, which are currently underway (NCT04614246).

In conclusion, eliapixant demonstrates selective blockade of the P2X3 receptor and is expected to have favorable efficacy, safety, and tolerability profiles suitable for long-term treatment in multiple indications, including those currently under-served by available management strategies (e.g., endometriosis). The high selectivity of eliapixant, in combination with its long half-life and hence low peak-trough ratio in humans, will determine the long-term usability of this compound, as a disturbed taste perception significantly impacts patients’ quality of life. In the context of endometriosis, blocking ATP-activated P2X3 receptors may prevent the cycle of neurogenic inflammation in addition to inhibiting nociceptive effects. Therefore, eliapixant could potentially offer a dual benefit in alleviating pain symptoms, including non-menstrual pelvic pain, and also modifying the underlying disease in endometriotic lesions. Eliapixant is currently under clinical development for endometriosis (NCT04614246), as well as other hypersensitive nerve fiber-associated disorders, including RUCC (NCT04562155), DNP (NCT04641273) and OAB (NCT04545580).

## Materials and methods

### Cell lines

The purinergic receptors P2X3 (human) and P2X2/3 (human and rat) were stably expressed in the human 1321N1 cell line, and the rat P2X3 was expressed in an inducible human HEK T-REx cell line (cells were obtained from Renovis Inc., cell line generation is described in detail in the patent WO 2009110985 A2^68^). Cells were maintained in Dulbecco’s Modified Eagle Medium (DMEM) media (Sigma) supplemented with 10% fetal bovine serum (FBS) (Sigma) in a humidified incubator with 5% CO_2_ at 37 °C.

### Drugs and reagents

The antagonist (eliapixant; BAY 1817080) was synthesized in-house, stored at 4 °C, and dissolved in dimethyl sulfoxide (DMSO) prior to use. The agonist, α,β-meATP, was obtained from Sigma. For in vivo* studies,* CFA (Complete Freund’s Adjuvant), urethane, mustard oil, and Evans blue were obtained from Sigma. Ibuprofen was obtained from Fluka Analytical.

### Calcium flux assay (FLIPR)

P2X receptor-induced changes in intracellular calcium levels were monitored using the calcium-chelating dye, Fluo-4AM (Molecular Probes), quantitated with a fluorescence imaging plate reader (FLEX/FLIPR station; Molecular Devices, Sunnyvale, CA, USA). Cells expressing P2X3 or P2X2/3 receptors were plated at a density of 15,000 cells/well in collagen-coated 384-well plates approximately 20 h before assay. On the day of the assay, 20 µL buffer (Hank's balanced salt solution, 20 mM HEPES, 0.5 mM CaCl_2_, 0.5 mM MgCl_2_, 0.1% bovine serum albumin [BSA], 5 mM probenecid, 10 mM d-glucose monohydrate, and 5 U/mL hexokinase, at pH 7.4) containing the 2 µM Fluo-4 AM dye was added to the cells and incubated for 90 min at 37 °C. The supernatant was then removed and replaced with 45 µL buffer without hexokinase. The eliapixant antagonist (5 µL) was added and allowed to incubate for 30 min at 37 °C. The final assay DMSO concentration was 1%. The agonist (α,β-meATP) was then added at a concentration representing the effective concentration (EC)_80_ value. The fluorescence was measured (excitation at 470–495 nm and emission at 515–575 nm) and analyzed based on the increase in peak relative fluorescence units (RFU) compared with the basal fluorescence. Peak fluorescence was used to determine the response to agonist obtained at each concentration of antagonist by the following equation: % Response = 100 * (RFU_drug_ − RFU_control_)/(RFU_DMSO_ − RFU_control_). Concentration–response curves were generated using Excel Fit software (Microsoft Office). Data were measured in triplicate and data from two different plates measured on different days were used to calculate the mean IC_50_ value.

### Whole-cell voltage patch-clamp assay using human recombinant P2X3 and P2X2/3 receptors

Experiments were performed under the whole-cell patch-clamp configuration at a holding potential of – 80 mV at room temperature. Cells expressing hP2X3 or hP2X2/3 receptors were grown to a confluence of 65–85% for ~ 20 h prior to assay. The cells were dissociated with Accumax, centrifuged at 1000 rpm for 3 min, re-suspended in manual patch-clamp buffer, and then plated in dishes at a density of ~ 50,000 cells/dish. The currents were recorded using HEKA amplifiers and Patchmaster software (HEKA Electronics). The bath solution contained 140 mM NaCl, 5 mM KCl, 1 mM CaCl_2_, 2 mM MgCl_2_, 25 mM HEPES, and 10 mM glucose, at pH 7.4. The intracellular solution contained 140 mM CsCl, 10 mM HEPES, and 10 mM BAPTA, at pH 7.0. Responses to an EC_80_ concentration of α,β-meATP agonist (10 µM for hP2X3 receptors and 30 μM for human P2X2/3 receptors) were allowed to stabilize during two to three agonist applications, and a rundown of ~ 20% was allowed between the last two applications. The recordings were performed in a cumulative manner with 4- or 8-min incubation times per compound concentration. Hexokinase (50 U/mL) was added to reduce channel desensitization when recording human and rat P2X3 receptor neurons. A seal resistance of minimum 250 MΩ and a series resistance of less than 20 MΩ were applied as quality control parameters. The effect at each concentration of antagonist was determined according to the following equation: % response = (100 * I_drug_)/I_control_), where I_control_ was the peak current amplitude of the last agonist addition prior to incubation with the antagonist, and I_drug_ was the peak current amplitude in the presence of an antagonist. Each concentration of antagonist was tested on a minimum of three independent cells. The concentration of drug required to inhibit P2X3 receptor current by 50% (IC_50_) was determined by fitting of the Hill equation to the averaged percent response data at each concentration in Excel Fit software.

### Whole-cell voltage patch-clamp assay in rat neurons

The DRG (L6–L4) and NDG were dissected from female Sprague Dawley rats and placed in cell culture medium DMEM (Gibco). The ganglia were de-sheathed, centrifuged, and resuspended in 400 µL of solution containing 0.5 mg/mL dispase, 2.5 mg/mL collagenase, 6 mg/mL BSA, and 10 mM HEPES. Cells were then incubated for 30 min, centrifuged, and washed twice with cultivation medium. The cultivation medium for DRG neurons contained neurobasal medium (GIBCO) supplemented with 10% FBS (Gibco), 250 ng/mL NGF (BD Bioscience), 2 mM l-Glutamine, 1X Supplement B-27 (Gibco), 200 IU/mL penicillin, and 200 µg/mL streptomycin. The cultivation medium for NDG neurons contained L15 medium (Gibco) supplemented with 10% FBS (Gibco), 50 ng/mL NGF (BD Bioscience), 0.2% sodium hydrogen carbonate, 5.5 mg/mL glucose, 200 IU/mL penicillin, 200 µg/mL streptomycin. The ganglia were then gently triturated 10 times with a large-bore fire-polished Pasteur pipette until they were dissociated, filtered through a 50 µm filter, centrifuged, and re-suspended in cultivation medium. Cells were plated a density of 1000 cells/well on poly-d-Lysine and laminin pre-coated coverslips placed in a 12-well plate. Electrophysiological measurements of cells from DRG neurons started 3–4 h after seeding, whereas the measurements of NDG neurons started the next day. Experiments were performed as described above for the recombinant cell lines using the α,β-meATP agonist (10 µM) to record DRG and NDG activity.

### Immunofluorescence staining of PX23 and nerve fibers in human endometrial tissue

Human endometriosis lesion tissue samples were obtained from provitro GmbH (Berlin, Germany). Patients provided informed consent for the provision of all tissue samples. Tissue samples were paraffin embedded, sectioned, and mounted on Superfrost slides. Prior to staining, mounted tissue sections were deparaffinized and rehydrated. Sections were then permeabilized in 0.3% Triton X-100/phosphate buffered saline (PBS) and blocked in 10% normal goat serum/PBS. Sections were then co-stained by incubating with the primary antibodies, α-P2X3 rabbit polyclonal (Abcam, ab10269; 1:1000) and α-PGP9.5 mouse monoclonal (Abcam, ab8189; 1:1000) diluted in 1% normal goat serum and 0.1% Triton X-100 in PBS at 4 °C overnight. Sections were washed three times in PBS for 15 min and incubated with appropriate Alexa Fluor secondary antibodies diluted in 3% normal goat serum for 2 h at room temperature. Sections were washed in PBS as described above and counterstained using DAPI to visualize nuclei. Mounted sections were dried overnight and coverslipped using Flouromount-G. Images were acquired with an Olympus IX81 microscope (10× objective, NA: 0.30, resolution: 0.92 μm, Olympus).

### Animals

Adult female and male Sprague Dawley rats (RjHan:SD; 175–200 g at delivery; Janvier Laboratories, Saint-Berthevin Cedex, France) and female C57BL/6N mice (Charles River, Sulzfeld, Germany, 10 weeks of age at delivery) were used in these experiments. For experiments performed in rats and mice, animals were obtained and acclimatized for at least 5 days before the start of a study. Rats were housed in a temperature (22 ± 2 °C)-—and humidity (55 ± 5%)—controlled room, maintained in a 12-h light/dark cycle with lights on at 06:00 AM. Rats were housed individually or in groups, and mice in groups, in transparent polycarbonate cages with clean standard bedding and environmental enrichment (paper towels and/or wood sticks), with ad libitum access to food and water. Animals studies were performed in accordance with ARRIVE guidelines.

### Rat hyperalgesia model

Mechanical hyperalgesia was induced in 32 healthy male Sprague Dawley rats by injecting 25 µL of CFA (1 mg/mL) unilateral into the plantar surface of one hind paw. Mechanical hyperalgesia was measured using the Pressure Application Measurement apparatus (Ugo Basile, Comerio, Italy). Briefly, a linearly increasing pressure was applied to an area of ~ 50 mm^2^ of the plantar side of the hind paw until a behavioral response (paw withdrawal) was observed, or until the pressure reached 1000-g force. The pressure at which the behavioral response occurred was recorded as the PWT. Both CFA-injected and contralateral PWTs were determined for each rat, in each treatment group, and at each time point of the study. Mechanical hyperalgesia measurement was performed before injecting CFA (pre-CFA), 22 h after CFA treatment (pre-dose baseline post-CFA), as well as at 2, 4, and 6 h after compound administration. Compounds were dosed 24 h after CFA treatment. Upon completion of the experiments, rats were euthanized by exposure to carbon dioxide. The study included four experimental groups of eight rats, including: three groups of rats treated with eliapixant at either 1, 3, or 10 mg/kg and one group of rats treated with the vehicle (0.5% carboxymethylcellulose [CMC] in water:Tween 80 [95:5, v/v], 5 mL/kg). All dose formulations were letter coded and administered to the animals by one technician not involved in conducting the behavioral PAM testing. All PAM measurements were performed by a second technician who was blinded to the treatments received by the animals.

### Mouse hyperalgesia model

Mechanical hyperalgesia was induced in female C57BL/6 mice by injecting 30 µL of undiluted CFA into the medial left plantar hind paw. This procedure was performed on anesthetized animals using inhaled isoflurane 48 h after CFA injections. Mice were placed individually in transparent Plexiglas enclosures set on a wire mesh floor for at least 60 min prior to testing. Mechanical response thresholds were determined using calibrated von Frey monofilaments applied to the plantar surface of hind paws. The simplified up-down method^69^ was used to estimate the 50% withdrawal threshold of contralateral and ipsilateral paws. Compounds were administered orally, twice daily, starting one hour before CFA injection, and included: eliapixant at 0.5 mg/kg, 5 mg/kg, and 25 mg/kg; ibuprofen at 100 mg/kg; and the vehicle, 0.5% CMC:Tween 80 (95:5, v/v). All dose formulations were letter coded and administered to the animals by a technician not involved in conducting the behavioral von Frey testing. All von Frey measurements were performed by a second experimenter who was blinded to the treatments received by the animals. Upon completion of the experiments, mice were euthanized by exposure to carbon dioxide.

### Rat neurogenic inflammation model

Twelve healthy female Sprague Dawley rats in pro-estrus phase were anesthetized with urethane (1.4 g/kg at a volume of 5 mL/kg intraperitoneal) and treated with an intravenous bolus injection of eliapixant (0.7 mg/kg), followed by an intravenous infusion of 0.2 mg/kg/h. Ten additional female rats were injected with vehicle control (0.3% DMSO/0.3% solutol in saline) following the same experimental design as compound-treated animals. Mustard oil (10% solution in mineral oil) was then injected into the left uterine horn (0.25 mL/rat), and 2-h later, Evans blue was injected intravenously. After 30 min, animals were killed by cardiac flushing with sterile saline. Uterine neurogenic inflammation was assessed by counting the number of blue dots on the ventral and dorsal sides in the abdominal region as an indicator of cutaneous neurogenic plasma extravasation following chemical uterine inflammation, as previously described^17^.

### Rat dyspareunia model

Dyspareunia was surgically induced in 32 female Sprague Dawley rats by autotransplanting on abdominal arteries small pieces of uterine horn, that grow into vascularized cysts. The VMR to vaginal distension was used in conscious animals as an objective measure of vaginal sensitivity. Briefly, mature healthy female rats in estrus phase were anesthetized with a mixture of 1:1 (v/v) ketamine (< 100 mg/mL)/Rompun (< 2%), injected intraperitoneally (up to 1 mL/kg). Biopsies of the left uterine horn were implanted around alternate cascade mesenteric arteries that supply the small intestine (four pieces in total, 4 × 4 mm in size) and on the wall of the distal colon (two pieces in total, 2 × 2 mm in size). In addition, to measure the VMR response, two Teflon-coated wire electrodes (AS632; Cooner Wire, USA) were sutured in the external oblique abdominal muscle, and then tunneled subcutaneously to be exteriorized at the base of the neck for future access. As analgesics, rats received flunixin (1 mg/kg at a volume of 5 mL/kg intraperitoneal) and tramadol (2.5 mL/100 L drinking water over 3 days) prior and after surgery, respectively. On the day of VMR assessment, a lubricated small balloon (embolectomy catheter 5F, 1 cm in length; IsoMed, France) was inserted into the mid-vaginal canal. The balloon catheter was secured to the base of the tail and connected to a volume controller/timing device (PHD Ultra syringe pump; Harvard Apparatus, United Kingdom) for balloon distension. The vaginal balloon was inflated using water to ramp intensities of distension (from 0 to 0.8 mL, by increments of 0.05 mL every 20 s [= 0.15 mL/min]). The electrodes were connected to an amplifier (Animal Bio Amp; ADInstruments, Germany), and the abdominal EMG signals were amplified and filtered (low pass 5000 Hz; high pass 10 Hz) using a data acquisition system (PowerLab; ADInstruments, Germany) for off-line analysis using LabChart version 7 (ADInstruments, Germany). The magnitude of the VMR at each vaginal distension test was quantified by counting manually the number of EMG spike bursts (or abdominal muscle contractions) per period of 40 s (= 0.1 mL distension volume). Upon completion of the experiments, rats were euthanized by exposure to carbon dioxide. Eliapixant or the vehicle (0.5% CMC:Tween 80 [95:5, v/v]) were dosed orally, twice daily (8 h apart), during two consecutive weeks, from week four to week five post-implantation of uterine horn biopsies. One experimental group (n = 17) was dosed with eliapixant at 15 mg/kg twice daily, and a second experimental group (n = 15) was dosed with vehicle at 5 mL/kg twice daily. VMR/vaginal distension testing were then performed at week five (on-drug) and week six (off-drug) post-implantation of uterine horn pieces, when the animals were in the pro-estrus phase. For each animal, the cumulative number of abdominal contractions was calculated and plotted against vaginal distension volume, and the corresponding AUC was calculated. The manual counting of the number of abdominal muscle contractions was performed by one technician who was blinded to the treatments received by the animals.

### Statistical analysis

All data are expressed as mean (or mean ± SD), unless otherwise indicated. Data were analyzed with either GraphPad Prism (v6.03, https://www.graphpad.com/scientific-software/prism/) or the Excel Fit software. For the rat hyperalgesia model, data were analyzed by computing the mean PWT for each treatment group, for CFA-injected and non-injected hind paws, and at each time point of the study. Mechanical hyperalgesia in vehicle control group was evaluated by carrying out an unpaired t-test between ipsilateral and contralateral paw values at each time point. The effect of the test compound on hyperalgesia was evaluated by performing a two-way repeated measures-ANOVA on ipsilateral paw values, followed by a Dunnett’s post-hoc test, provided that a main effect for treatment was detected. The effect of the test compound on contralateral response was evaluated following the same statistical procedure. For the rat neurogenic inflammation and dyspareunia models, data were analyzed by computing the mean number of skin dots or AUCs, respectively, for each treatment group and at each time point of the study (where appropriate). A non-parametric Mann–Whitney U test was then used to compare compound-treated group versus vehicle control. A Grubbs’ test was also performed on individual values to reveal potential outliers. A p-value of < 0.05 was considered statistically significant.

### Ethical approval

All animal studies were carried out in strict accordance with Bayer AG and Evotec SE policies, AAALAC guidelines and the European Directive 2010/63/EU as well as the German Animal Welfare Act (Tierschutzgesetz, TierSchG). All animal experiments were approved by the Institutional Animal Care and Use Committee (IACUC) and competent regional Animal Care and Use Committees according to §15 TierSchG (Berlin and Hamburg, Germany).

## Data Availability

The majority of the data generated or analyzed during this study are included in this published article. Remaining data are available from the corresponding author on reasonable request.

## Supplementary﻿ Information

Supplementary Information.
