# Supplementary material for: Eliapixant is a selective P2X3 receptor antagonist for the treatment of disorders associated with hypersensitive nerve fibers
Source: Sci Rep. 2021 Oct 6;11:19877. doi: 10.1038/s41598-021-99177-0 (PMC8494816; doi:10.1038/s41598-021-99177-0)

Supplementary Material

Supplemental Table 1: Clinical trials involving eliapixant (BAY 1817080).

Indication	Clinical Phase	Clinical Trial Identifier
Refractory and/or unexplained chronic	IIb	NCT04562155
Overactive bladder	IIa	NCT04545580
Endometriosis	IIb	NCT04614246
Diabetic neuropathic pain	IIA	NCT04641273

Supplemental Figure 1: Chemical structure of eliapixant (BAY 1817080).

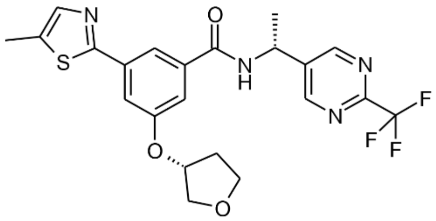

Supplemental Figure 2: Representative FLIPR traces for eliapixant (BAY 1817080) against recombinant 1321N1 cell lines expressing human P2X3 and human P2X2/3 (top panels) as well as rat P2X3 and rat P2X2/3 (bottom panels) in a fluorescence imaging plate reader (FLIPR) calcium-flux assay for human P2X3 and human P2X2/3.

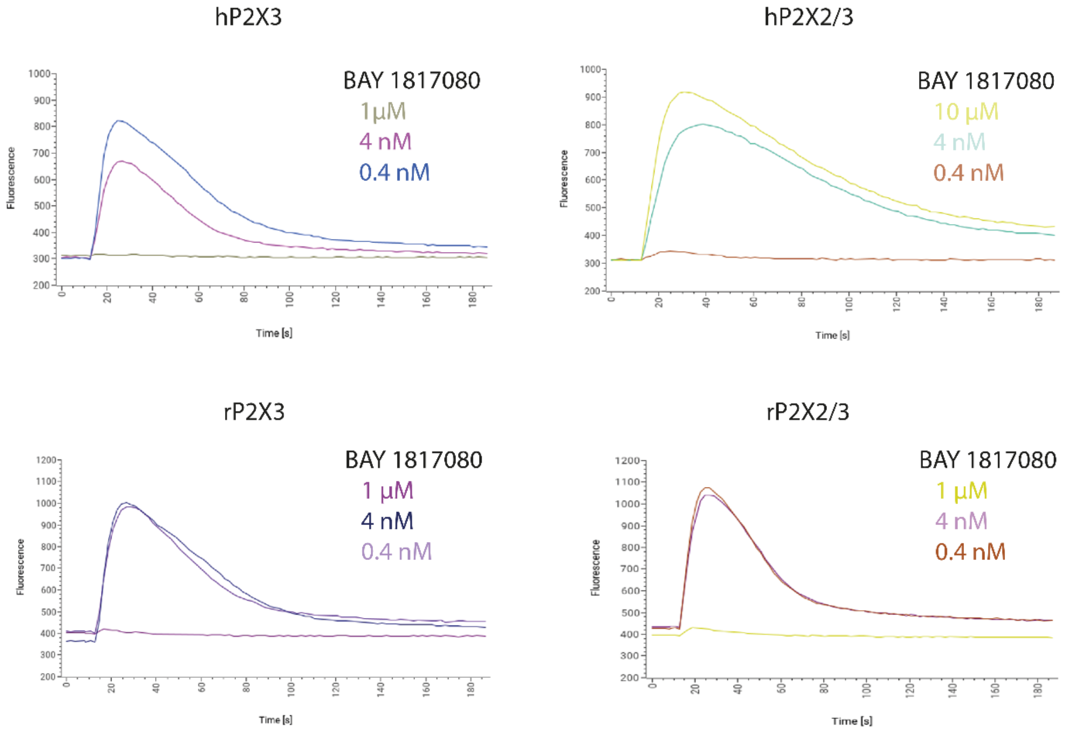

Supplement: Supplementary file 1 — Supplementary Information. [file 41598_2021_99177_MOESM1_ESM.pdf]
